# Building the future of ICU care: Is our digital foundation strong enough? A multicentre survey of Australian and New Zealand intensive care units

**DOI:** 10.1016/j.ccrj.2025.100133

**Published:** 2025-10-17

**Authors:** Kristen S. Gibbons, Renate Le Marsney, Andrew Goodwin, Rayna Reddy, Patricia Gilholm, David Pilcher, Ben Gelbart, the Australian and New Zealand Intensive Care Society Paediatric Study Group (ANZICS PSG)

**Affiliations:** a Children's Intensive Care Research Program, Child Health Research Centre, The University of Queensland, Brisbane, QLD, Australia; b School of Biomedical Engineering, University of Sydney, Sydney, NSW, Australia; c The University of Queensland Medical School, Brisbane, QLD, Australia; d Alfred Health, Melbourne, VIC, Australia; e The Australian and New Zealand Intensive Care Society (ANZICS) Centre for Outcome and Resource Evaluation, Prahran, VIC, Australia; f Australian and New Zealand Intensive Care Research Centre, School of Public Health and Preventive Medicine, Monash University, VIC, Australia; g Paediatric Intensive Care Unit, Royal Children's Hospital, Parkville, VIC, Australia; h Clinical Sciences, Murdoch Children's Research Institute, Parkville, VIC, Australia

**Keywords:** Intensive care, Digital health, Electronic health record, Health informatics

## Abstract

**Objectives:**

The objective of this study was to assess data-related resources, infrastructure, and capabilities in Australia and New Zealand (ANZ) intensive care units (ICUs).

**Design:**

Electronic multicentre survey was conducted.

**Setting:**

ANZ ICUs between June and October 2024.

**Participants:**

All ANZ ICUs contributing to the Australian and New Zealand Intensive Care Society Adult Patient Database and/or Australian and New Zealand Paediatric Intensive Care Registry were included in this study.

**Interventions:**

There are none to declare.

**Main outcome measures:**

The main outcome measures included types of medical records, digital data capture and research availability, digital enhancement plans, staffing, and research collaboration.

**Results:**

Of 209 ICUs, 112 (54%) responded; 13 paediatric, 21 mixed, and 78 adult ICUs, with responses from all ANZ jurisdictions. Overall, 59% used paper records (5 paediatric and 61 mixed/adult), 28% digitised (7 paediatric and 24 mixed/adult), and 59% electronic health records (EHRs; 10 paediatric and 56 mixed/adult), with most EHRs introduced within the last decade (76%). In units with an EHR, 59% collected data secondly or minutely in the EHR and >75% collected EHR data on patient demographics, clinical notes, laboratory results, medications, fluids, bedside monitors, and respiratory support devices. Data Managers were employed within 45% of ICUs, with 96% able to extract data for audit and 92% for research. Respondents reported frustrations with delayed EHR implementation and limited data extraction mechanisms.

**Conclusions:**

Substantial variability exists across ANZ ICUs in digital health adoption, data capture, and data management resources. Quantifying differences in digital information, improving data extraction, and building collaborative networks are key steps for supporting research and innovation across units.

## 1 Introduction

The ongoing digitisation of health represents a profound shift in the healthcare landscape.^1^ The transition from traditional paper records to digital formats, such as electronic health records (EHRs), has streamlined the way patient information is documented, accessed, and shared. This transformation not only enhances the efficiency of healthcare delivery but also enables collaboration among healthcare providers, leading to more informed decision-making.[2], [3], [4] Digital health technologies extend beyond records, encompassing a spectrum of innovations such as telemedicine, wearables, and health apps, empowering individuals to actively participate in their wellbeing.^5^^,^^6^ The digitisation of health not only promises improved patient outcomes through data-driven insights but also lays the foundation for a more connected, patient-centric, and technologically advanced healthcare ecosystem.^7^

However, the advantages associated with such advances are only harnessed with dedicated resourcing, infrastructure, and capabilities. The expertise required are broad, ranging from timely extraction of granular data from complex EHRs, analysis of data to drive quality improvement (QI) and research, and the resulting implementation and translation of findings into clinical practice. Accessing and amalgamating EHR data alone can present significant challenges, particularly when dealing with diverse record systems across multiple sites.^8^ Distinct record-keeping practices and incompatible formats hamper seamless data extraction and collaborative efforts.^9^ Overcoming these barriers necessitates robust local infrastructure capable of supporting data curation, extraction, and analysis, as well as co-ordinated and harmonised efforts across institutions. Establishing such infrastructure ensures interoperability, enabling the integration of diverse datasets to derive meaningful insights and enhance the quality of patient care.

Simultaneously, the rise of artificial intelligence (AI)–assisted clinical decision-making marks a transformative era. Achieving the successful development and translation of AI tools into clinical practice hinges on the availability of well-curated data and highly skilled analysts partnering with clinicians^10^ and development of a robust ethical framework for the assessment of models before they are deployed clinically.^11^ To date, in Australia and New Zealand (ANZ), there has been limited use of the data-rich intensive care unit (ICU) environment to boost the generation of knowledge to support precision medicine. However, the ICU presents a unique opportunity to generate high-quality AI-assisted clinical decision-making approaches; large quantities of biosamples can be collected, EHRs are often available, and extensive digitised physiological monitoring exists. To harness this potential, local, national, and international collaborations are key, ensuring a high volume of records from diverse populations. To date, there is no documented knowledge on the current landscape of data-related resources, infrastructure, and capabilities across ANZ to guide binational collaborative research in this area.

As such, we aimed to assess the existing data-related resources, infrastructure, and capabilities in ANZ that could underpin future research collaborations to leverage AI-assisted clinical decision-making within the ICU environment.

## 2 Methods

We report the study according to the Checklist for Reporting Results of Internet E-Surveys.^12^ Ethical approval was received from The University of Queensland Faculty of Medicine Low Risk Human Research Ethics Committee (2024/HE000285).

An open electronic survey was distributed via e-mail to all ANZ ICUs who contribute to the Australian and New Zealand Paediatric Intensive Care Registry or Adult Patient Database, hosted by the Australian and New Zealand Intensive Care Society Centre for Outcome and Resource Evaluation (ANZICS CORE). This sampling frame accounts for 92% (209/226) of ANZ ICUs. The survey was developed by the study team, following a review of the literature where no suitable or similar surveys were available. We assessed the face validity by requesting colleagues in different professional roles and with differing levels of experience to undertake the survey. The survey was refined based on this feedback (Supplemental File 2) and implemented in Research Electronic Data Capture (REDCap; hosted by The University of Queensland^5^^,^^6^).

The survey was sent to the Director, Nurse Unit Manager, and data collector of each eligible unit, with two follow-up reminders sent. One response per unit was requested, with the unit identifying the most appropriate staff member to complete the survey. Responses were identifiable to permit clarification of responses where required. Participants were informed of the approximate length of time to complete the survey, where data would be stored, who the investigators were, and the purpose of the study prior to survey commencement. Completion and submission of the survey was taken as implied consent. Participants were given the option to directly report the number of ICU beds, staffing resources, and the number of admissions or to allow linkage of data from their response to ANZICS data collected through the annual ANZICS CORE Critical Care Resources survey that is completed by the same ICUs. All respondents agreed to linking relevant Critical Care Resources data. Participation in the survey was voluntary, and there were no incentives offered to participate. Survey responses were stored securely and only accessed by the study team.

The survey consisted of 80 questions organised into three content sections (Participant Information [relating to respondent and unit information], Records [including paper records, digitised records which are those that have been converted from physical to digital representations via scanning or photography, and EHRs], and Resourcing and Infrastructure) and questions relating to site contacts and interest in collaborative research (Supplement 2). Many questions were conditionally displayed based on responses to other questions. Respondents could change any responses up until the point of survey submission. The consistency and completeness of survey responses were assessed by the study team. Sites with duplicate or incomplete responses were followed up by the study team, and responses were updated and/or consolidated accordingly.

The response rates are presented as a number and proportion. Data relating to categorical variables (e.g., yes/no) are presented as number and proportion; continuous data are summarised as median, interquartile range (IQR), and/or range. Graphical representation of data through use of bar charts is presented to aid interpretation. As the survey questions were not mandatory, some missing data persisted even after follow-up. No units were excluded due to missing data, and as such, the denominator is presented in text and tables where necessary. Free-text responses were reviewed by the study team and allocated to themes if possible and are presented descriptively. Units and individuals are not identified in publication of results. As this is a convenience sample with results presented descriptively, no sample size calculation was undertaken. Analyses were undertaken in StataSE version 18.0 (StataCorp Pty Ltd, College Station, Texas).

## 3 Results

The survey was distributed to 209 ICUs between June and October 2024. A total of 112 units responded (54% response rate); 13 (of 13, 100% response rate) paediatric, 21 (of 25, 84% response rate) mixed, and 78 (of 171, 46% response rate) adult ICUs, with responses from all ANZ jurisdictions (Table 1). The survey was completed by nurse unit managers (39/112; 35%), directors (34/112; 30%), data managers/researchers (16/112; 14%), and other clinical roles (21/112; 19%). Public ICUs responded more frequently than private ICUs (87/137; 64% vs 25/72; 35%). Units that responded had a median 13 (IQR: 9, 21; range: 4, 62) physical beds and 850 (IQR: 542, 1457; range: 71, 3443) annual admissions (Table 1).

**Table 1:** Response rates, characteristics of survey respondents, and comparison of characteristics between ICUs with and without an electronic health record (EHR).

Characteristic	Number of units surveyed	Response rate n (%)	No EHR (N = 46) n (%)	EHR (N = 66) n (%)
Overall	209	112 (54%)	46 (41%)	66 (59%)
**ICU type**^a^				
Paediatric	13	13 (100%)	3 (23%)	10 (77%)
Mixed	25	21 (84%)	9 (43%)	12 (57%)
Adult	171	78 (46%)	34 (44%)	44 (56%)
**Hospital funding model**^a^				
Private	72	25 (35%)	23 (92%)	2 (8%)
Public	137	87 (64%)	23 (26%)	64 (74%)
**ICU regionality**^a^				
Metropolitan	152	79 (52%)	35 (44%)	44 (56%)
Regional/rural	57	33 (58%)	11 (33%)	22 (67%)
**CICM level**^a^**^,#^**				
1	32	14 (44%)	7 (50%)	7 (50%)
2	89	40 (45%)	20 (50 %)	20 (50 %)
3 (including PICUs)	88	58 (66%)	19 (33%)	39 (67%)
**State**^a^				
Australian Capital Territory	5	2 (40%)	1 (50%)	1 (50%)
New South Wales	62	30 (48%)	6 (20%)	24 (80%)
New Zealand	21	12 (57%)	8 (67%)	4 (33%)
Northern Territory	2	2 (100%)	2 (100%)	0
Queensland	41	22 (54%)	8 (36%)	14 (64%)
South Australia	12	7 (58%)	4 (57%)	3 (43%)
Tasmania	5	2 (40%)	2 (100%)	0
Victoria	48	29 (60%)	12 (41%)	17 (59%)
Western Australia	13	6 (46%)	3 (50%)	3 (50%)
**Unit respondent characteristics**		**N** = **112**	**N** = **46**	**N** = **66**
**Total physical ICU beds**^b^		13 (9, 21)	10 (8, 17)	14 (10, 24)
**Total available ICU beds**^b^		10 (7, 17)	10 (8, 16)	10 (7, 22)
**Total yearly admissions**^b^		850 (542, 1457)	785 (538, 1341)	857 (560, 1732)
**Is there a data manager role within your ICU?**^c^				
No		62 (55%)	40 (87%)	22 (33%)
Yes		50 (45%)	6 (13%)	44 (67%)
**How is the data manager funded?**^d^		N = 50	N = 6	N = 44
Permanent funding		46 (92%)	5 (83%)	41 (93%)
Temporary funding		6 (12%)	1 (17%)	5 (11%)
**Data manager FTE**^c^		N = 50	N = 6	N = 44
<1.0		26 (52%)	4 (67%)	22 (50%)
≥1.0		23 (46%)	2 (33%)	21 (48%)
Unknown		1 (2%)	0	1 (2%)
**What other data-related roles within, or outside, your ICU does your unit engage?**^d^				
None		44 (39%)	26 (57%)	18 (27%)
Biomedical engineer		28 (25%)	9 (20%)	19 (29%)
Data manager		23 (21%)	4 (9%)	19 (29%)
Data engineer		17 (15%)	4 (9%)	13 (20%)
Data scientist		14 (13%)	1 (2%)	13 (20%)
Other		17 (15%)	5 (11%)	12 (18%)

# Brief descriptions of CICM levels: level 1 ICU, “… capable of providing immediate resuscitation and short term cardio-respiratory support …”, level 2 ICU, “… capable of providing a high standard of general intensive care, including complex multisystem life support …”, level 3 ICU, “… tertiary referral unit for intensive care patients and should be capable of providing comprehensive critical care including complex multisystem life support for an indefinite period”, PICU, “… capable of providing comprehensive critical care including complex multisystem life support for an indefinite period to children less than 16 years”.^13^ CICM: College of Intensive Care Medicine; ICU: intensive care unit; IQR: interquartile range; FTE: full-time equivalent; PICU: paediatric intensive care unit.

a Row percentages.

b Median (IQR).

c n (%).

d Multiple responses can be chosen.

### 3.1 Types of records

**Fig. 1:**
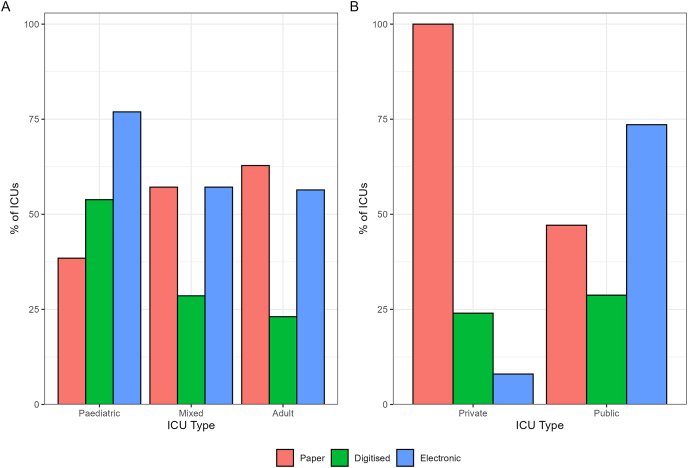
Types of records used in Australian and New Zealand intensive care units (ICUs), by A) ICU patient population and B) hospital funding model.

The same number of units reported using paper and/or electronic records (59% each), with 28% of ICUs using digitised records (Table 2; Fig. 1). Of the 66 units using an electronic record, 61% (40/66) are using an electronic record exclusively, while 26 are using a hybrid approach, including 12 supplementing the electronic record with a paper record, four with a digitised record, and 10 with both paper and digitised records (Table 2). Common characteristics of ICUs with an EHR were paediatric units, public units, located in New South Wales or Queensland, and tertiary referral units (level 3 units, including paediatric ICUs, as designated by the College of Intensive Care Medicine^13^; Table 1). For ICUs without an EHR (N = 46), nine (20%) are planning to introduce an EHR into the ICU (seven in 2025 and two in 2026), 31 (67%) are not planning to introduce an EHR, and the remaining six are unsure. Respondents without an EHR commented that the significant financial cost associated with implementing an EHR was an ongoing barrier, despite a strong desire at both ICU and hospital levels for EHRs. The majority of EHRs were introduced within the past 10 years (76%) with 59% specific to the ICU (Table 3).

**Table 2:** Data-related resources and infrastructure across Australian and New Zealand intensive care units (N = 112).

Resources/infrastructure	N = 112
**Medical record type**^a^	
Paper	66 (59%)
Digitised	31 (28%)
Electronic	66 (59%)
**Medical record type**	
Paper only	29 (26%)
Digitised only	2 (2%)
Electronic only	40 (36%)
Paper + digitised	15 (13%)
Digitised + electronic	4 (4%)
Paper + electronic	12 (11%)
Paper + digitised + electronic	10 (9%)
**Is any ICU waveform data collected and stored?**	
No	98 (88%)
Yes	5 (4%)
Do not know	9 (8%)
**Are physiological measurements stored in any parallel systems (other than the EHR)?**	
No	83 (74%)
Yes	17 (15%)
Don't know	12 (11%)
**What other data-related resources/infrastructure within, or outside, your ICU does your unit use?**^a^	
None	39 (35%)
Data collection systems separate to EHR	37 (33%)
Data transfer/sharing platforms between sites	25 (22%)
Other	9 (8%)

a Multiple responses can be chosen; EHR: electronic health record; ICU: intensive care unit.

**Table 3:** Additional characteristics of electronic health records (EHRs) across Australian and New Zealand intensive care units (N = 66).

EHR Characteristic	N = 66
**What year did the EHR start? (N** = **61)**	
<2010	5 (8%)
2010–2014	10 (16%)
2015–2019	26 (43%)
2020–2024	20 (33%)
**Is the EHR specific to the ICU, or used across the whole hospital?**	
Specific to the ICU	39 (59%)
Used across the whole hospital	27 (41%)
**Does the EHR allow access from external computer programs for data extraction?**	
No	13 (20%)
Yes	32 (48%)
Do not know	21 (32%)
**Who can extract the data using external computer programs? (N** = **32)**	
Only the data manager(s) for the EHR	12 (38%)
Anyone with specific credentials	14 (44%)
Anyone with access to the EHR	1 (3%)
Other	2 (6%)
Do not know	3 (9%)
**What is the most granular measurement frequency of data collected in the EHR?**	
Secondly	5 (8%)
Minutely	34 (52%)
5 minutely	2 (3%)
30 minutely	2 (3%)
Hourly	5 (8%)
Other	4 (6%)
Do not know	14 (21%)

EHR: electronic health record; ICU: intensive care unit.

### 3.2 Resources and infrastructure

Almost half of the units employed a data manager (50/112; 45%), predominantly appointed with permanent funding (46/50; 92%). Units without a data manager often reported that nursing staff were filling the duties of a data manager. EHR-enabled units were more likely to employ a data manager (67% vs 13%) as well as engage with additional data-related roles, including biomedical engineers (29%), data scientists, or data engineers (20% each). Respondents also reported engaging with a range of other data-related roles (17/112) within and outside their units, including hospital-wide information technology and business intelligence, quality departments with access to various data repositories, and health department application specialists.

### 3.3 Data recording and extraction

**Fig. 2:**
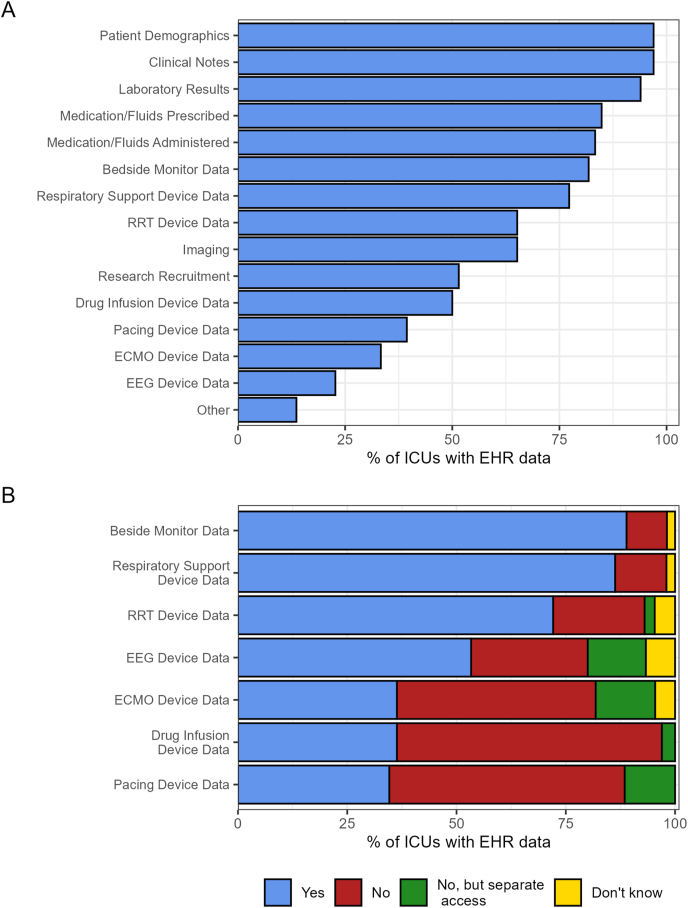
Overview of characteristics of electronic health records (EHRs; N = 66). A) Types of data collected in the EHR. B) Data flow from external devices into the EHR. ECMO: extracorporeal membrane oxygenation; EEG: electroencephalogram; EHR electronic health record; ICU: intensive care unit; RRT: renal replacement therapy.

The most common data collected in EHRs included patient demographics, clinical notes, laboratory results, medication/fluids prescribed, medication/fluids administered, bedside monitor data, and respiratory support devices (each collected in >75% of EHRs) (Fig. 2). For the 26 units that use hybrid approaches including an EHR, the most common data collected electronically (collected in >75% of EHRs) are patient demographics, clinical notes, and laboratory results. Most of these data flow automatically into the EHR, particularly bedside monitor data (89%), respiratory support device data (86%), and renal replacement therapy data (72%) (Fig. 2). Data are collected secondly or minutely in 59% of units with an EHR. Waveform data are rarely collected and stored (5/112; 4%), as are physiological measurements stored in parallel systems outside of the EHR (17/112; 15 %). Data managers can extract data for audit/clinical review (48/50; 96%), research (46/50; 92%), and system improvements (40/50; 80%). External access to EHRs for the purpose of data extraction is available for 48% (32/66) of ICUs and for a range of users (Table 1).

## 4 Discussion

In this binational survey of ANZ ICUs, 59% of ICUs reported operating an EHR of which 61% are exclusively electronic. EHRs are predominantly available in public hospitals, compared to private ones, and are more common in paediatric hospitals than in adult hospitals; although this could be in part due to highest response rates in these cohorts. Demographic, clinical, laboratory, and device data were commonly recorded data types, whereas waveform data storage was uncommon. Only two thirds of units with an EHR employ a data manager supporting clinical audit, research, and system improvement, potentially limiting the utility of EHR data for research and quality assurance activities.

Our reported rate of 59% EHR adoption aligns with that of other high-income countries such as the United States^14^ and Canada,^15^ who are incentivised by policies such as the “meaningful use” initiative.^14^ However, ANZ ICUs face a mixed landscape relating to EHR benefits: while comprehensive EHRs improve data accessibility^16^ and patient safety,^17^ their impact on clinical outcomes, including mortality reduction, remains inconsistent.^18^^,^^19^ The disparity in EHR maturity across regions,^20^ particularly in resource-constrained settings,^21^^,^^22^ positions ANZ as digitally progressive but not yet fully optimised. This is further highlighted through differences in digital health use and data management between public and private ICUs. Public ICUs had a higher response rate (64% vs 35%) and were much more likely to have an EHR (74% vs 8%). This could be due to public hospitals in ANZ benefiting from centralised mandates and government funding,^23^ facilitating research participation and digital adoption. Conversely, in low- and middle-income countries, where an “infrastructure inequality trap” often disproportionately directs government funding towards private rather than public hospitals,^24^ private ICUs tend to have higher research participation due to greater access to resources and decision-making autonomy. These differences emphasise the need to consider funding and organisational factors when evaluating and planning for digital health adoption.

To further progress ANZ’s digital adoption, globally recognised enablers and barriers of EHR implementation should be considered. Robust infrastructure, effective training, and user-centric system design are key identified enablers. Stable internet connectivity,^20^ reliable hardware,^17^ and interoperability^25^ are foundational for EHR functionality and integration. Specialised, hands-on training alongside experiential learning improves EHR adoption and reduces resistance.^17^ A user-centric system design^20^—focused on workflow integration, intuitive interfaces, and customisation for ICU needs—ensures that digital tools integrate into ICU workflows without adding to clinicians’ cognitive and administrative burdens.^26^ Barriers to EHR adoption include financial constraints, poor system design, and clinician burnout. High implementation costs,^27^ maintenance expenses,^28^ and usability issues^17^^,^^25^ hinder uptake, particularly in low-resource settings.^20^ Increased documentation demands^20^ contribute to cognitive overload, burnout, and dissatisfaction.^17^^,^^27^ Addressing these barriers requires strategic investments in funding, system usability, and clinician well-being to ensure sustainable EHR integration to accelerate data-driven ICU research.

Research readiness utilising data accessed through EHR systems relies on high-quality data. Such quality can be assessed in the categories of intrinsic data quality, contextual data quality, representational data quality, and accessible data quality.^29^ However, to date, there is no agreed-upon standard or framework for assessing EHR data quality, hampering reliability of research using such data.^30^ Additionally, emerging ICU research increasingly focuses on machine learning models that track patient and model risk in near real time.^31^ Development and testing of these models requires timely access to high-frequency data[32], [33], [34]; however, ANZ ICUs primarily collect low-frequency data. Fewer than 10% of sites report capturing signals with a frequency of once per second, and only 4% reported collecting clinical waveform data such as electroencephalograms, electrocardiograms, or photoplethysmograms. The power of data-driven research is underpinned by large datasets; aggregating and harmonising data across multiple hospitals is crucial for multicentre research,^35^^,^^36^ but variability in secondary data collection methods complicates this.^37^^,^^38^ Ideally, hospitals should collect patient data prospectively and uniformly, with automated tools enabling near–real time access. Systems lacking programmatic access to recently collected data impede the development, testing, and deployment of real-time clinical models in ICUs. Without access to continuous, high-resolution physiological data, harmonised across multiple sites and optimised for research, the development, testing, and implementation of real-time predictive models remain significantly constrained.

Despite the research potential of high-frequency physiological data, ethical and governance frameworks often restrict its use. Clinical data are collected without explicit consent and stored in the EHR, while access to data for research requires consent (or a waiver of such) and is often stored separately. This distinction between “clinical” and “research” data impacts data accessibility and limits the potential for comprehensive physiological databanks.^39^ High-frequency signals, particularly physiological waveforms collected from bedside monitors, are typically stored outside the EHR as part of research projects rather than classified as clinical data. As a result, they are not subject to the same legal retention requirements or backup protocols as clinical data stored in the EHR and are unlikely to be collected continuously or proactively without patient consent. While numerous databanks of physiological signals have been established and released publicly,[40], [41], [42], [43], [44], [45] none of these currently include ANZ patients, highlighting a missed opportunity for ANZ-informed data-driven advancements. Addressing these ethical and governance challenges is critical to unlocking the full potential of ICU data for research and AI-driven innovations.

In addition to research, high-quality data infrastructure is integral to ICU QI initiatives. ICUs with robust EHRs can efficiently track key performance indicators, audit practices, and implement data-informed interventions. However, reliance on paper records or inconsistent electronic systems hampers real-time evaluations and benchmarking.^46^ The capacity to extract and analyse structured, high-resolution data is fundamental to continuous improvement cycles, particularly in identifying and addressing preventable harms. Strengthening data-related infrastructure across all ICUs will enhance research capabilities and patient care, requiring a national commitment to comprehensive, interoperable digital systems.

While our survey has provided insights into an otherwise underexplored area, it is not without limitations. The response rates differed between sectors, most prominently between public (64%) and private (35%) sectors, and between paediatric (100%) and adult (46%) ICUs (noting there is some relationship between these two characteristics as in ANZ all paediatric ICUs are publicly funded). As such, there is potential for response bias, resulting in potential for the EHR adoption rate to be overestimated given the higher response rates from sectors more likely to have EHRs. Additionally, there was no existing validated tool that was fit for purpose. We developed our survey specifically for this project and would encourage others to use and adapt the survey for their own region.

Achieving a robust, interoperable digital infrastructure is essential for advancing data-driven research and QI in ANZ ICUs. While progress has been made in EHR adoption, the coexistence of paper, electronic, and hybrid record systems highlights the ongoing transition to digital health care and introduces challenges in data accessibility, standardisation, and interoperability. For research collaborations reliant on high-quality, standardised data, this variability represents a significant barrier.^47^ Addressing these disparities requires targeted investment in infrastructure, standardised data-sharing frameworks, and policies that support real-time, high-resolution data collection. A coordinated approach that promotes digital adoption while ensuring interoperability between existing systems is essential.^48^ Investment in data-sharing frameworks that enable secure, national initiatives promoting common data structures, and governance models could facilitate seamless data integration, unlocking AI-driven predictive analytics, real-time benchmarking, and precision medicine applications.^49^^,^^50^ Without decisive action, current fragmentation will continue to limit data-driven advancements in critical care.

## CRediT authorship contribution statement

KSG designed the study and wrote the protocol in conjunction with RL, AG, PG, DP and BG. Data analyses were performed by KSG, RL and PG. The first draft of the manuscript was written by KSG, RL, AG, RR, PG and BG, and all authors commented and critically revised the manuscript. All authors approved the final manuscript.

## Funding

KSG is supported by a National Health and Medical Research Council Investigator Grant (Australia). BG is supported by a Clinician Scientist Fellowship at the Murdoch Children’s Research Institute Fellowship (Australia). The funding sources have had no involvement in study design, analyses, or interpretation or reporting of the results.

## Conflict of interest

The authors declare the following financial interests/personal relationships which may be considered as potential competing interests: Ben Gelbart declares they are part of the *Critical Care and Resuscitation* editorial team as an Associate Editor. If there are other authors, they declare that they have no known competing financial interests or personal relationships that could have appeared to influence the work reported in this paper.

## Appendix B

**The Australian and New Zealand Intensive Care Society Paediatric Study Group**

Tatjana Kerig, Australian and New Zealand Paediatric Intensive Care Registry; Jennifer Darvas, Marino Festa, The Children’s Hospital at Westmead, Sydney, Australia; Shane George, Gold Coast University Hospital, Gold Coast, Australia; Corrine Balit, John Hunter Hospital, Newcastle, Australia; Felix Oberender, Monash Children’s Hospital, Melbourne, Australia; Simon Erickson, Jenipher Chumbes Flores, Perth Children’s Hospital, Perth, Australia; Karina Charles, Sainath Raman, Michaela Waak, Tara Williams, Queensland Children’s Hospital, Brisbane, Australia; Debbie Long, Queensland University of Technology, Brisbane, Australia; Warwick Butt (Chair), Ben Gelbart, Kate Masterson, Johnny Millar, Royal Children’s Hospital, Melbourne, Australia; Anusha Ganeshalingam, Claire Sherring, Starship Children’s Hospital, Auckland, New Zealand; Puneet Singh, Vicky Smith, Sydney Children’s Hospital, Randwick, Australia; Kristen S Gibbons (Vice-Chair), Luregn J Schlapbach (Past Chair), Jessica Schults, The University of Queensland, Brisbane, Australia.
