# Supplementary material for: Building the future of ICU care: Is our digital foundation strong enough? A multicentre survey of Australian and New Zealand intensive care units
Source: Crit Care Resusc. 2025 Oct 17;27(4):100133. doi: 10.1016/j.ccrj.2025.100133 (PMC12554109; doi:10.1016/j.ccrj.2025.100133)
Supplement: Multimedia component 1 [file mmc1.docx]

***Supplementary materials to:***

**Building the future of ICU care: Is our digital foundation strong enough? A multicentre survey of Australian and New Zealand intensive care units**

Kristen S Gibbons^1^, Renate Le Marsney^1^, Andrew Goodwin^2^, Rayna Reddy^1,3^, Patricia Gilholm^1^, David Pilcher^4,5,6^, Ben Gelbart^7,8^ on behalf of the Australian and New Zealand Intensive Care Society Paediatric Study Group (ANZICS PSG)

^1^ Children’s Intensive Care Research Program, Child Health Research Centre, The University of Queensland, Brisbane, Australia

^2^ School of Biomedical Engineering, University of Sydney, Sydney, Australia

^3^ The University of Queensland Medical School, Brisbane, Australia

^4^ Alfred Health, Melbourne, Victoria, Australia

^5^ The Australian and New Zealand Intensive Care Society (ANZICS) Centre for Outcome and Resource Evaluation, Prahran, Victoria, Australia.

^6^ Australian and New Zealand Intensive Care Research Centre, School of Public Health and Preventive Medicine, Monash University

^7^ Paediatric Intensive Care Unit, Royal Children’s Hospital, Parkville, Victoria, Australia

^8^ Clinical Sciences, Murdoch Children’s Research Institute, Parkville, Victoria, Australia

**Corresponding author:**

Professor Kristen Gibbons

Children’s Intensive Care Research Program

Child Health Research Centre

Faculty of Health, Medicine, and Behavioural Sciences

The University of Queensland

62 Graham St

South Brisbane Q 4101

+61 407 966 708

[k.gibbons@uq.edu.au](mailto:k.gibbons@uq.edu.au)

**Table S1**. Additional characteristics of electronic health records across Australian and New Zealand Intensive Care Units (N=66).

| **EHR Characteristic** | **N=66** |
| --- | --- |
| **What vendor is used for the ICU EHR? (N=39)** |  |
| Vendor 1 | 33 (85%) |
| Vendor 3 | 3 (8%) |
| In-house | 2 (5%) |
| Vendor 2 | 1 (3%) |
| **What vendor is used for the whole hospital EHR? (N=27)** |  |
| Vendor 2 | 12 (44%) |
| Vendor 5 | 4 (15%) |
| Vendor 4 | 3 (11%) |
| Vendor 6 | 2 (7%) |
| Vendor 7 | 2 (7%) |
| Other | 3 (11%) |
| Don’t know | 1 (4%) |
| **What data are collected in the EHR?*** |  |
| Patient Demographics | 64 (97%) |
| Clinical Notes | 64 (97%) |
| Laboratory Results | 62 (94%) |
| Medication/Fluids Prescribed | 56 (85%) |
| Medication/Fluids Administered | 55 (83%) |
| Bedside Monitor Data | 54 (82%) |
| Respiratory Support Device Data | 51 (77%) |
| RRT Device Data | 43 (65%) |
| Imaging | 43 (65%) |
| Research Recruitment | 34 (52%) |
| Drug Infusion Device Data | 33 (50%) |
| Pacing Device Data | 26 (39%) |
| ECMO Device Data | 22 (33%) |
| EEG Device Data | 15 (23%) |
| Other | 9 (14%) |
| **Does bedside monitor data flow automatically into the EHR?** | **N=54** |
| No | 5 (9%) |
| Yes | 48 (89%) |
| Don’t know | 1 (2%) |
| **Does respiratory support device data flow automatically into the EHR?** | **N=51** |
| No | 6 (12%) |
| Yes | 44 (86%) |
| Don’t know | 1 (2%) |
| **Does RRT device data flow automatically into the EHR?** | **N=43** |
| No, but can be accessed separately | 1 (2%) |
| No | 9 (21%) |
| Yes | 31 (72%) |
| Don’t know | 2 (5%) |
| **Does ECMO device data flow automatically into the EHR?** | **N=22** |
| No, but can be accessed separately | 3 (14%) |
| No | 10 (45%) |
| Yes | 8 (36%) |
| Don’t know | 1 (5%) |
| **Does EEG device data flow automatically into the EHR?** | **N=15** |
| No, but can be accessed separately | 2 (13%) |
| No | 4 (27%) |
| Yes | 8 (53%) |
| Don’t know | 1 (7%) |
| **Does pacing device data flow automatically into the EHR?** | **N=26** |
| No, but can be accessed separately | 3 (12%) |
| No | 14 (54%) |
| Yes | 9 (35%) |
| **Does drug infusion device data flow automatically into the EHR?** | **N=33** |
| No, but can be accessed separately | 1 (3%) |
| No | 20 (61%) |
| Yes | 12 (36%) |

* multiple responses can be chosen; EHR electronic health record; ICU Intensive Care Unit; RRT renal replacement therapy; ECMO extracorporeal membrane oxygenation; EEG electroencephalogram
