# Supplementary material for: Building the future of ICU care: Is our digital foundation strong enough? A multicentre survey of Australian and New Zealand intensive care units
Source: Crit Care Resusc. 2025 Oct 17;27(4):100133. doi: 10.1016/j.ccrj.2025.100133 (PMC12554109; doi:10.1016/j.ccrj.2025.100133)
Supplement: Multimedia component 2 [file mmc2.pdf]

Data Capability Survey

ANZICS PSG

Data Capability Survey

Thank you for your interest in completing our survey.

Survey Information:
You are invited to participate in a research project assessing the existing data-related resources, infrastructure, and capabilities in Australian and New Zealand Intensive Care Units.

The transition from traditional paper records to digital formats, such as electronic health records (EHRs), has streamlined the way patient information is documented, accessed, and shared. This transformation not only enhances the efficiency of healthcare delivery but also enables collaboration among healthcare providers, leading to more informed decision-making. The digitisation of health is projected to lead to improved patient outcomes through data-driven insights, but also lay the foundation for a more connected, patient-centric, and technologically advanced healthcare ecosystem. The data rich ICU environment presents a unique opportunity to generate high-quality AI-assisted clinical decision-making approaches. To harness this potential, local, national, and international collaborations are key, ensuring a high volume of records from diverse populations. To date, there is no documented knowledge on the data-related resources, infrastructure, and capabilities across Australia and New Zealand to guide binational collaborative research in this area.

Research Team:
A/Prof Kristen Gibbons (Principal Investigator), Children's Intensive Care Research Program (ChIRP), Child Health Research Centre, The University of Queensland, Australia
Dr Ben Gelbart, Paediatric Intensive Care Unit, Royal Children's Hospital Melbourne
Dr Andrew Goodwin, School of Biomedical Engineering, University of Sydney
Dr Paula Lister, Paediatric Critical Care Unit, Sunshine Coast University Hospital
Renate Le Marsney, ChIRP
Trang Pham, ChIRP
Dr Marino Festa, Paediatric Intensive Care Unit, Children's Hospital Westmead
Dr Trish Gilholm, ChIRP
Dr Johnny Millar, Paediatric Intensive Care Unit, Royal Children's Hospital Melbourne
A/Prof Debbie Long, School of Nursing, Faculty of Health, Queensland University of Technology
Kate Masterson, Paediatric Intensive Care Unit, Royal Children's Hospital Melbourne
Prof David Pilcher, Department of Intensive Care, Alfred Health

This survey has been endorsed by the Australian and New Zealand Intensive Care Society Paediatric Study Group, through the Data Science Working Group.

Survey Instructions:

This online survey should take approximately 15 minutes to complete. Participation is voluntary, and we are seeking one response per hospital. We are asking for your name and organisation to ensure we don't have duplicate responses per hospital, and to enable further contact if we need to clarify any responses. If you don't know the answer to a question, please feel free to seek out further input from a colleague. You can choose to skip any questions you don't want to answer. Participation or non-participation will not impact your relationship with any members of the research team, or the organisations involved in the Study. Submission of the survey will be interpreted as your implied consent to participate and that you affirm that you are at least 18 years of age. This Study adheres to the guidelines of the ethical review process of The University of Queensland and the National Statement on Ethical Conduct in Human Research (2007, updated 2023). Whilst you are free to discuss your participation in this Study with project staff (contactable on +617 3069 7253 or email k.gibbons@uq.edu.au), if you would like to speak to an officer of the University not involved in the study, you may contact the Ethics Coordinators on +617 3365 3924 / +617 3443 1656 or email humanethics@research.uq.edu.au.

PARTICIPANT INFORMATION

Date of Survey Commencement:

____
(DD-MM-YYYY)

Name:

____

Position:

____

Email:

____

ICU Name:

- ◯ Albury Wodonga Health ICU
- ◯ Alfred Hospital ICU
- ◯ Alice Springs Hospital ICU
- ◯ Angliss Hospital ICU
- ◯ Armadale Health Service ICU
- ◯ Ashford Community Hospital ICU
- ◯ Auckland City Hospital CV ICU
- ◯ Auckland City Hospital DCCM
- ◯ Austin Hospital ICU
- ◯ Ballarat Health Services ICU
- ◯ Bankstown-Lidcombe Hospital ICU
- ◯ Bathurst Base Hospital ICU
- ◯ Bendigo Health Care Group ICU
- ◯ Blacktown Hospital ICU
- ◯ Bowral Hospital HDU
- ◯ Box Hill Hospital ICU
- ◯ Braemar Hospital SCU
- ◯ Brisbane Private Hospital ICU
- ◯ Broken Hill Base Hospital & Health Services ICU
- ◯ Buderim Private Hospital ICU
- ◯ Bunbury Regional Hospital ICU
- ◯ Bundaberg Base Hospital ICU
- ◯ Caboolture Hospital ICU
- ◯ Cabrini Hospital ICU
- ◯ Cairns Hospital ICU
- ◯ Calvary Adelaide Hospital ICU
- ◯ Calvary Bruce Private Hospital HDU
- ◯ Calvary Hospital (Lenah Valley) ICU
- ◯ Calvary John James Hospital ICU
- ◯ Calvary Mater Newcastle ICU
- ◯ Calvary North Adelaide Hospital ICU
- ◯ Campbelltown Hospital ICU
- ◯ Canberra Hospital ICU
- ◯ Casey Hospital ICU
- ◯ Central Gippsland Health Service (Sale) ICU
- ◯ Christchurch Hospital ICU
- ◯ Coffs Harbour Health Campus ICU
- ◯ Concord Hospital (Sydney) ICU
- ◯ Dandenong Hospital ICU
- ◯ Dubbo Base Hospital ICU
- ◯ Dunedin Hospital ICU
- ◯ Echuca Regional Hospital HDU
- ◯ Epworth Eastern Private Hospital ICU
- ◯ Epworth Freemasons Hospital ICU
- ◯ Epworth Geelong ICU
- ◯ Epworth Hospital (Richmond) ICU
- ◯ Fairfield Hospital ICU
- ◯ Fiona Stanley Hospital ICU
- ◯ Flinders Medical Centre ICU
- ◯ Flinders Private Hospital ICU
- ◯ Footscray Hospital ICU
- ◯ Frankston Hospital ICU
- ◯ Gold Coast Private Hospital ICU
- ◯ Gold Coast University Hospital ICU
- ◯ Gold Coast University Hospital PICU
- ◯ Gosford Hospital ICU
- ◯ Gosford Private Hospital ICU
- ◯ Goulburn Base Hospital ICU
- ◯ Goulburn Valley Health ICU
- ◯ Grafton Base Hospital ICU
- ◯ Grampians Health Horsham ICU
- ◯ Greenslopes Private Hospital ICU
- ◯ Griffith Base Hospital ICU
- ◯ Hawkes Bay Hospital ICU
- ◯ Hervey Bay Hospital ICU
- ◯ Hollywood Private Hospital ICU
- ◯ Holmesglen Private Hospital ICU
- ◯ Hornsby Ku-ring-gai Hospital ICU
- ◯ Hurstville Private Hospital ICU

◯ Hutt Hospital ICU
◯ Ipswich Hospital ICU
◯ John Fawkner Hospital ICU
◯ John Flynn Private Hospital ICU
◯ John Hunter Children's Hospital PICU
◯ John Hunter Hospital ICU
◯ Joondalup Health Campus ICU
◯ Kareena Private Hospital ICU
◯ Knox Private Hospital ICU
◯ Latrobe Regional Hospital ICU
◯ Launceston General Hospital ICU
◯ Lingard Private Hospital ICU
◯ Lismore Base Hospital ICU
◯ Liverpool Hospital ICU
◯ Logan Hospital ICU
◯ Lyell McEwin Hospital ICU
◯ Mackay Base Hospital ICU
◯ Macquarie University Private Hospital ICU
◯ Maitland Hospital ICU
◯ Maitland Private Hospital ICU
◯ Manning Rural Referral Hospital ICU
◯ Maroondah Hospital ICU
◯ Mater Adults Hospital (Brisbane) ICU
◯ Mater Private Hospital (Brisbane) ICU
◯ Mater Private Hospital (Sydney) ICU
◯ Mater Private Hospital (Townsville) ICU
◯ Melbourne Private Hospital ICU
◯ Middlemore Hospital ICU
◯ Mildura Base Public Hospital ICU
◯ Monash Children's Hospital PICU
◯ Monash Medical Centre-Clayton Campus ICU
◯ Mount Hospital ICU
◯ Mount Isa Hospital ICU
◯ Mulgrave Private Hospital ICU
◯ National Capital Private Hospital ICU
◯ Nelson Hospital ICU
◯ Nepean Hospital ICU
◯ Nepean Private Hospital ICU
◯ Newcastle Private Hospital ICU
◯ Noosa Hospital ICU
◯ North Canberra Hospital ICU
◯ North Shore Hospital ICU
◯ North Shore Private Hospital ICU
◯ North West Regional Hospital (Burnie) ICU
◯ Northeast Health Wangaratta ICU
◯ Northern Beaches Hospital ICU
◯ Norwest Private Hospital ICU
◯ Orange Base Hospital ICU
◯ Peninsula Private Hospital ICU
◯ Perth Children's Hospital PICU
◯ Pindara Private Hospital ICU
◯ Port Macquarie Base Hospital ICU
◯ Prince of Wales Hospital (Sydney) ICU
◯ Prince of Wales Private Hospital (Sydney) ICU
◯ Princess Alexandra Hospital ICU
◯ Queen Elizabeth II Jubilee Hospital ICU
◯ Queensland Children's Hospital PICU
◯ Redcliffe Hospital ICU
◯ Robina Hospital ICU
◯ Rockhampton Hospital ICU
◯ Rockingham General Hospital ICU
◯ Rotorua Hospital ICU
◯ Royal Adelaide Hospital ICU
◯ Royal Brisbane and Women's Hospital ICU
◯ Royal Children's Hospital (Melbourne) PICU
◯ Royal Darwin Hospital ICU
◯ Royal Hobart Hospital ICU
◯ Royal Hobart Hospital NICU/PICU
◯ Royal Melbourne Hospital ICU
◯ Royal North Shore Hospital ICU
◯ Royal Perth Hospital ICU

◯ Royal Prince Alfred Hospital ICU
◯ Ryde Hospital and Community Health Services ICU
◯ Shoalhaven Hospital ICU
◯ Sir Charles Gairdner Hospital ICU
◯ South East Regional Hospital ICU
◯ South West Healthcare (Warrnambool) ICU
◯ Southern Cross Hospital (Hamilton) ICU
◯ Southern Cross Hospital (Wellington) ICU
◯ St Andrew's Hospital (Adelaide) ICU
◯ St Andrew's Hospital Toowoomba ICU
◯ St Andrew's Private Hospital (Ipswich) ICU
◯ St Andrew's War Memorial Hospital ICU
◯ St George Hospital (Sydney) ICU
◯ St George Private Hospital (Sydney) ICU
◯ St John Of God Health Care (Subiaco) ICU
◯ St John Of God Hospital (Ballarat) ICU
◯ St John of God Hospital (Bendigo) ICU
◯ St John of God Hospital (Berwick) ICU
◯ St John Of God Hospital (Geelong) ICU
◯ St John Of God Hospital (Murdoch) ICU
◯ St John of God Midland Public & Private ICU
◯ St Vincent's Private Hospital Northside ICU
◯ St Vincent's Hospital (Melbourne) ICU
◯ St Vincent's Hospital (Sydney) ICU
◯ St Vincent's Hospital (Toowoomba) ICU
◯ St Vincent's Private Hospital (Sydney) ICU
◯ St Vincent's Private Hospital Fitzroy ICU
◯ Starship Children's Hospital PICU
◯ Sunnybank Hospital ICU
◯ Sunshine Coast University Hospital ICU
◯ Sunshine Coast University Hospital PICU
◯ Sunshine Coast University Private Hospital ICU
◯ Sunshine Hospital ICU
◯ Sutherland Hospital & Community Health Services ICU
◯ Sydney Adventist Hospital ICU
◯ Sydney Children's Hospital PICU
◯ Sydney Southwest Private Hospital ICU
◯ Tamworth Base Hospital ICU
◯ Taranaki Health ICU
◯ Tauranga Hospital ICU
◯ The Children's Hospital at Westmead PICU
◯ The Chris O'Brien Lifehouse ICU
◯ The Memorial Hospital (Adelaide) ICU
◯ The Northern Hospital ICU
◯ The Prince Charles Hospital ICU
◯ The Queen Elizabeth (Adelaide) ICU
◯ The Wesley Hospital ICU
◯ Timaru Hospital ICU
◯ Toowoomba Hospital ICU
◯ Townsville University Hospital ICU
◯ Townsville University Hospital PICU
◯ Tweed Heads District Hospital ICU
◯ University Hospital Geelong ICU
◯ Victorian Heart Hospital ICU
◯ Wagga Wagga Base Hospital & District Health ICU
◯ Waikato Hospital ICU
◯ Warringal Private Hospital ICU
◯ Wellington Hospital ICU
◯ Werribee Mercy Hospital ICU
◯ Western District Health Service (Hamilton) ICU
◯ Western Hospital (SA) ICU
◯ Westmead Hospital ICU
◯ Westmead Private Hospital ICU
◯ Whakatane Hospital ICU
◯ Whangarei Area Hospital - Northland Health Ltd ICU
◯ Wollongong Hospital ICU
◯ Wollongong Private Hospital ICU
◯ Women's and Children's Hospital PICU
◯ Wyong Hospital ICU

Do you consent for ANZICS to provide the research team with the number of beds, admissions and staffing resources for your unit?

Collected through the Annual ANZICS Critical Care Resources Survey

○ Yes ○ No

Number of physical beds in your ICU:

____

Total number of admissions per year to your ICU:

____

Total number of paediatric admissions (patients < 16 years of age) per year to your ICU:

____

RECORDS

Please answer questions in relation to the current state of records/clinical information systems in your ICU

Type(s) of medical record in your ICU:

Select all that apply.

'Digital Records' = These are digitised versions of physical documents or documents originally created in a digital format. This could be a scanned paper form or a Word document created on a computer.

'Electronic Records' = These records are borne and exist solely within computer systems.

☐ Paper Record
☐ Digitised Record
☐ Electronic Health Record (EHR)

What year did digitisation start?

____
([XXXX])

Has optimal character recognition (OCR) been used to digitise into a readable format?

'OCR' = The use of technology to distinguish printed or handwritten text characters inside digital images of physical documents, such as a scanned paper document.

○ Yes
○ No
○ Don't know

What year did the EHR start?

____
([XXXX])

Is the EHR specific to the ICU, or used across the whole Hospital?

○ Specific to the ICU
○ Used across the whole Hospital
○ Don't know

What vendor is used for the ICU EHR?

○ MetaVision
○ Epic
○ Cerner
○ Other
○ Don't know

Please specify "Other" vendor used for the ICU EHR:	__________________________
What vendor is used for whole Hospital EHR?	◯ Epic<br>◯ Cerner<br>◯ Other<br>◯ Don't know
Please specify "Other" vendor used for whole Hospital EHR:	__________________________
What data is collected in the EHR?<br><br>Select all that apply.	☐ Bedside Monitor Data<br>☐ Respiratory Support Device Data<br>☐ RRT Device Data<br>☐ ECMO Device Data<br>☐ EEG Device Data<br>☐ Pacing Device Data<br>☐ Drug Infusion Device Data<br>☐ Laboratory Results<br>☐ Imaging<br>☐ Medication/Fluids Prescribed<br>☐ Medication/Fluids Administered<br>☐ Patient Demographics<br>☐ Clinical Notes<br>☐ Research Recruitment<br>☐ Other
Please describe the "Other" EHR data collected.	________________________________
Does bedside monitor data flow automatically into the EHR?	◯ Yes<br>◯ No<br>◯ No, but can be accessed separately<br>◯ Don't know
Does respiratory support device data flow automatically into the EHR?	◯ Yes<br>◯ No<br>◯ No, but can be accessed separately<br>◯ Don't know
Does RRT device data flow automatically into the EHR?	◯ Yes<br>◯ No<br>◯ No, but can be accessed separately<br>◯ Don't know
Does ECMO device data flow automatically into the EHR?	◯ Yes<br>◯ No<br>◯ No, but can be accessed separately<br>◯ Don't know
Does EEG device data flow automatically into the EHR?	◯ Yes<br>◯ No<br>◯ No, but can be accessed separately<br>◯ Don't know

Does pacing device data flow automatically into the EHR?	◯ Yes<br>◯ No<br>◯ No, but can be accessed separately<br>◯ Don't know
Does drug infusion device data flow automatically into the EHR?	◯ Yes<br>◯ No<br>◯ No, but can be accessed separately<br>◯ Don't know
What is the most granular measurement frequency of data collected in the EHR?	◯ Secondly<br>◯ Minutely<br>◯ 5-minutely<br>◯ 15-miutely<br>◯ 30-minutely<br>◯ Hourly<br>◯ Other<br>◯ Don't know
Please specify "Other" measurement frequency:	__________________________________
Is more frequent data stored in any systems outside the EHR?	◯ Yes<br>◯ No<br>◯ Don't know
Please outline the type and frequency of any data that is stored more frequently outside the EHR:	__________________________________
What brand/s of bedside monitors are used in ICU?<br><br>Select all that apply.	☐ Phillips<br>☐ GE<br>☐ Mind Ray<br>☐ Nihon Kohden<br>☐ Drager<br>☐ Other<br>☐ Don't know
Please specify "Other" brand of bedside monitor used in the ICU:	__________________________________
Is any ICU waveform data collected and stored? e.g. ventilator waveform data?	◯ Yes<br>◯ No<br>◯ Don't know
Please outline the type/s of ICU waveform data that is collected and stored:	__________________________________
Are physiological measurements stored in any parallel systems other than the EHR in ICU?	◯ Yes<br>◯ No<br>◯ Don't know
What parallel system is used?	____________________________

What is the primary purpose of the parallel system?<br><br>Select all that apply.	☐ Clinical<br>☐ Research<br>☐ Don't know
Is data from all, some, or no ICU beds collected in the parallel system?	◯ All beds<br>◯ Some beds<br>◯ No beds<br>◯ Don't know
Please outline how many beds have this type of data collection, how they are chosen, and whether there are plans to expand data collection.	__________________________________
Does the EHR allow access from external computer programs for data extraction? e.g. using a Python/SQL script	◯ Yes<br>◯ No<br>◯ Don't know
Who can extract the data using external computer programs?	◯ Only the Data Manager/s for the EHR<br>◯ Anyone with specific credentials<br>◯ Anyone with access to the EHR<br>◯ Other<br>◯ Don't know
Please specify "Other" person/s who can extract data using external computer programs:	____________________________
Is the hospital introducing an EHR into the ICU?	◯ Yes<br>◯ No<br>◯ Don't know
What year do they plan to introduce the EHR?	____________________________<br>([XXXX])
What vendor do they plan to use for the EHR?	◯ MetaVision<br>◯ Epic<br>◯ Cerner<br>◯ Other<br>◯ Don't know
Please specify "Other" vendor they plan to use for the EHR:	____________________________
Please outline any other plans to enhance electronic data collection in your unit:	__________________________________

RESOURCING & INFRASTRUCTURE
Please answer questions in relation to the current state of resources and infrastructure in your ICU

Is there a Data Manager role within your ICU?<br><br>'Data Manager' = Person employed to oversee the organisation, validation, reporting and secure storage of digital data. This does not include persons who enter data into the EHR/other data collection systems	◯ Yes<br>◯ No<br>◯ Don't know
How are they funded?<br><br>Select all that apply.	☐ Permanent funding<br>☐ Temporary funding. e.g. by research grants<br>☐ Don't know
Data Manager FTE:	____________________________
What can they extract data for?<br><br>Select all that apply.	☐ Audit/Clinical review<br>☐ Research<br>☐ ANZPICR<br>☐ System improvement<br>☐ Don't know
What other data-related roles within, or outside, your ICU does your unit engage with?<br><br>Select all that apply.	☐ Data Manager<br>☐ Data Scientist (person employed to analyse and interpret digital data)<br>☐ Data Engineer (person employed to build data pipelines to bring together information for different source systems)<br>☐ Biomedical Engineer<br>☐ Other<br>☐ None<br>☐ Don't know
Who is the Data Manager/s outside your ICU affiliated with?<br><br>Select all that apply.	☐ Hospital-based Health Information Management Systems (HIMS) team<br>☐ University<br>☐ Research Institute<br>☐ Commercial<br>☐ Other<br>☐ Don't know
Please specify "Other" affiliation for the Data Manager:	__________________________________
Who is the Data Scientist/s within, or outside, your ICU affiliated with?<br><br>Select all that apply.	☐ Employed within ICU<br>☐ Hospital-based Health Information Management Systems (HIMS) team<br>☐ University<br>☐ Research Institute<br>☐ Commercial<br>☐ Other<br>☐ Don't know
Please specify "Other" affiliation for the Data Scientist:	__________________________________

Who is the Data Engineer/s within, or outside, your ICU affiliated with?<br><br>Select all that apply.	☐ Employed within ICU<br>☐ Hospital-based Health Information Management Systems (HIMS) team<br>☐ University<br>☐ Research Institute<br>☐ Commercial<br>☐ Other<br>☐ Don't know
Please specify "Other" affiliation for the Data Engineer:	__________________________________
Who is the Biomedical Engineer/s within, or outside, your ICU affiliated with?<br><br>Select all that apply.	☐ Employed within ICU<br>☐ Hospital-based Health Information Management Systems (HIMS) team<br>☐ University<br>☐ Research Institute<br>☐ Commercial<br>☐ Other<br>☐ Don't know
Please specify "Other" affiliation for the Biomedical Engineer:	__________________________________
Please describe the "Other" data-related role/s within, or outside, your ICU.	__________________________________
What other data-related resources / infrastructure within, or outside, your ICU does your unit use?<br><br>Select all that apply.	☐ Data Collection Systems Separate to the EHR<br>☐ Data Transfer / Sharing Platforms between Sites<br>☐ Other<br>☐ None<br>☐ Don't know
Please specify "Other" data-related resources/infrastructure within, or outside your ICU that your unit uses:	__________________________________
Types of Data Collection Systems Used?<br><br>Select all that apply.	☐ REDCap<br>☐ Other
Please specify "Other" Types of Data Collection Systems:	__________________________________
Which tasks in REDCap can your site access support for?<br><br>Select all that apply.	☐ Project creation<br>☐ Database development<br>☐ Ongoing database maintenance<br>☐ No support<br>☐ Don't know

KEY SITE CONTACTS

Who could provide more information about data capability at your Site, or would have an interest in engaging further on this topic?

Select all that apply.

- ☐ Myself
- ☐ Health Information Management Systems (HIMS) Contact, or equivalent, e.g. Digital Health Services
- ☐ Other Site Contact(s)

HIMS Contact (or equivalent), Name: __________________________________

HIMS Contact (or equivalent), Position: __________________________________

HIMS Contact (or equivalent), Email: __________________________________

#01 Other Contact, Name: __________________________________

#01 Other Contact, Position: __________________________________

#01 Other Contact, Email: __________________________________

#02 Other Contact, Name: __________________________________

#02 Other Contact, Position: __________________________________

#02 Other Contact, Email: __________________________________

ADDITIONAL INFORMATION

The role of the Australia and New Zealand Intensive Care Society (ANZICS) Paediatric Study Group (PSG) Data Science Working Group is to further research capacity using data science and EHRs.

Data Science Working Group Aims:
To understand the evolving landscape of electronic data capture and storage in PICUs in ANZ.To understand the data science infrastructure in PICUs in ANZ.To understand the existing feasibility and develop methods for harmonising data extraction and collection across sites.To identify the infrastructure requirements to conduct such research.To identify study themes/studies that may be enabled by improved data science capability.To investigate opportunities to further leverage the ANZPIC Registry data for clinical trials and other data science projects.

Do you have an interest in joining the ANZICS PSG Data Science Working Group?

○ Yes ○ No

Would you like to receive a summary of the survey results?

○ Yes ○ No

GENERAL COMMENTS

Thank you for completing this survey.

Please use this space for any further comments you would like to make about your responses or about data capability at your Site: ______________________
